# "Raising a Ghost It Were Better to Lay"

**Published:** 1917-10-20

**Authors:** C. O. Hawthorne

**Affiliations:** Harley Street W.


### THE EDITOR'S LETTER-BOX.

### "Raising a Ghost it were Better to Lay."

*To the Editor of* The Hospital.

Sir,—Surely the writer of the article which appears under this title in your issue of the 13th inst. cultivates a curious fashion of public discussion. A certain medical organisation has advanced or supported the proposition that when patients who are pensioners of the State are admitted to hospitals payment should be made not only for their maintenance but also for the professional services rendered to them. It is, of course, open to anyone to argue either for or against this proposition. The writer of your article does neither the one nor the other, though he proposes the theme as the subject of his writing. He tells us that he would "prefer" to discuss an entirely different proposition—namely, that the medical and surgical staffs of our voluntary hospitals should be paid for the attendance they give to the ordinary hospital population; that is, to the sick poor. Of course, if the matter is one of taste or preference, there is nothing more to be said, for a journalist, like the rest of mankind, is free to cultivate whatever likes or dislikes best accommodate themselves to his own æsthetic disposition. But when, after defining his own inclination, he proceeds to suggest that those who advance the first of the two propositions stated above are endeavouring to "side-track" the second proposition, that is, the one more pleasing to his personal taste, he is in essence striving to regulate the judgment of others by the standard of his own individual fancifulness.

It is quite within the limits of reason to hold that services given to the State or to those for whom the State is responsible should be paid for, and yet to favour the continuance of gratuitous service to the sick poor. The two positions are by no means in conflict, and the arguments respectively relevant to them are far from being identical. Hence, your leader writer, while quite free to follow whatever course he "greatly likes," cannot be permitted without challenge to attempt to prejudice the position of those who rest their opinions on some more steady foundation than the "preference" favoured by an individual inclination. With the virtues of any one of these several propositions I am, at least for the moment, not concerned. My sole object is to protest that the two issues here in question must not be confused, and to claim the right to support the one without being censured for an endeavour to "side-track" the other. This claim may possibly hurt your correspondent's "tastes," but such an unhappy result must be credited to the attempt to erect these into an order and standard of reasoning. They have their value, I am sure. But their place is other than in the world of logic.

One other point. The article with which I am here concerned accuses a section of the medical profession of the ambition to "milk" a certain public authority, and "to make hay while the sun shines" in reference to possible payments for professional work, while it is good enough to characterise as "an exaggeration" a charge of "grab" which has been put into circulation by one of your contemporaries. That offensive statements of this order should be cultivated in quarters which need not here be particularised is a small affair, but to find them in a journal which may boast honourable professional associations, and which must know full well how freely the medical profession generally is ready to render gratuitous service where sickness and hardship are in question, is not a little surprising. In any event, it ought surely to be possible to argue whether State service should or should not receive State pay without the suggestion of sordid motives in those who uphold the affirmative of this proposition.—I am, Sir, yours faithfully,

Harley Street, W., C. O. Hawthorne.

October 12, 1917.

[Apart from its chaff, the object of Dr. Hawthorne's letter is to claim that payment for professional services to State pensioners who are admitted to voluntary hospitals is one proposition, and that payment for professional attendance on "the ordinary hospital population; that is, the sick poor," is another. We agree. But what are his reasons for suggesting that a desire to proceed from the first to the second was not influencing the British Medical Association in its recent policy? If it had no ulterior motive in advancing the first proposition, why did it seek to persuade the Ministry of Pensions to make the grant for the cost of maintenance of discharged men in voluntary hospitals include professional attendance, while leaving the War Office grant for the cost of maintenance of undischarged men severely alone? We suggested an explanation. It is for Dr. Hawthorne, who disputes its sufficiency, to suggest another. No doubt he can. Will he not do so?—The Writer of the Article.]

